# Phosphatase and Tensin Homolog (PTEN) Expression as a Surrogate Biomarker Correlated With the Depth of Invasion in Cutaneous Malignant Melanoma

**DOI:** 10.7759/cureus.45295

**Published:** 2023-09-15

**Authors:** Ziyi Sun, Hilal Arnouk

**Affiliations:** 1 Pathology, Midwestern University Chicago College of Osteopathic Medicine, Downers Grove, USA; 2 Pathology, Midwestern University College of Dental Medicine, Downers Grove, USA; 3 Pathology, Midwestern University Chicago College of Optometry, Downers Grove, USA; 4 Molecular Pathology, Midwestern University Precision Medicine Program, Downers Grove, USA

**Keywords:** tumor suppressor gene, pten, computer-assisted image analysis, histopathology and immunohistochemistry, tumor invasion, tnm classification, precision medicine oncology, prognostic biomarker, diagnostic biomarker, cutaneous malignant melanoma

## Abstract

Objective

The aim of this study is to evaluate the expression of the phosphatase and tensin homolog (PTEN), which is a tumor suppressor gene that is implicated in the pathogenesis of cutaneous malignant melanoma, in normal skin and melanoma tissue samples. The study also aimed to correlate PTEN expression levels with various clinicopathological parameters of melanoma lesions, thus highlighting the utility of PTEN expression as a prognostic biomarker for melanoma.

Study design

Immunohistochemistry (IHC) staining was performed on tissue microarray samples representing normal skin and melanoma biopsies of different clinicopathological parameters. Tissue photomicrographs were evaluated with Aperio ImageScope, which has a positive-pixel-counting algorithm built in. Subsequently, a histochemical score (H-score) was derived from the percentage of positive cells (%-staining) and their staining intensity. The H-scores were averaged in groups of tissue samples representing the different melanomas' tumor (T), node (N), and distant metastasis (M), also known as TNM parameters, as set forth by the American Joint Committee on Cancer (AJCC) classification. The mean H-scores were statistically compared using a two-tailed unpaired t-test.

Results

The PTEN protein expression was measured by IHC and found to be correlated with tumor thickness (T), which is a reliable indicator for survival rates. Specifically, PTEN was significantly downregulated in tumors with a thickness over 2 mm (T3+T4) compared to tumors with a thickness at or below 2 mm (T1+T2).

Conclusions

The PTEN protein expression, as measured by immunohistochemistry, helped differentiate between tumors with a thickness over 2 mm and tumors with a thickness at or below 2 mm, suggesting PTEN as a potential surrogate marker for the melanoma's invasion depth along with possible prognostic implications. Longitudinal studies evaluating risk stratification based on the expression of PTEN are needed to establish the utility of this promising biomarker in the clinic as an adjunct for pathological examination.

## Introduction

Cutaneous melanoma is the leading cause of death from skin cancer [1], even though it is the third most common skin cancer [2]. In predominantly fair-skinned, developed countries, such as the United States, cutaneous melanoma incidence has grown over 320% since 1975 [3]. Several risk factors contribute to the increase in the incidence rate [4]. They include host and environmental risk factors. Host risk factors such as family history, the number of congenital and acquired melanocytic nevi, and genetic susceptibility play a significant role in the development of melanoma [5]. More importantly, environmental risk factors such as ultraviolet sun radiation exposure and the use of artificial tanning beds have become more prevalent over the last few decades [6]. These risk factors, along with the lack of use of sun protection factor (SPF) screens, have contributed to the rise in the incidence rate of cutaneous melanoma.

Approximately 10% of melanoma cases are diagnosed at an advanced stage and are unresectable or are already metastatic at the time of diagnosis [5]. Malignant melanoma has a five-year survival rate of only 5% once the tumor has metastasized to distant tissues of the human body at the time of diagnosis [7]. Therefore, early detection of melanoma has several advantages, including reducing the size and extent of surgical removal, preventing long-term side effects associated with aggressive systemic treatments, and minimizing overall care costs [8]. Currently, diagnosis of cutaneous melanoma is based on histopathological examination, and prognosis for melanoma patients relies heavily on the TNM classification and clinical staging. Therefore, there is an unmet need for diagnostic and prognostic molecular biomarkers for malignant cutaneous melanoma.

The aim of the current investigation is to explore the prognostic utility of the phosphatase and tensin homolog (PTEN) as molecular biomarkers for cutaneous melanoma progression. The PTEN is an established tumor suppressor that is encoded by the PTEN gene on chromosome 10q23 and encodes for a protein with 403 amino acids [9]. Deletions or mutations of PTEN, leading to the loss of its enzymatic activity, contribute to enhanced cell proliferation, decreased cell death, and the promotion of tumor development [10]. The loss and downregulation of PTEN activity have been implicated in several malignancies, including breast, endometrial, prostate, ovarian, melanoma, colorectal, and lung cancers [11].

In this study, we have documented that the downregulation of PTEN expression is correlated with melanoma tissue thickness, which reflects the tumor depth of invasion. We hypothesize that the expression levels of PTEN correlate with the vertical depth of invasion (thickness) of cutaneous melanoma, which closely correlates with survival rates for melanoma patients. Thus, PTEN protein expression may serve as a surrogate biomarker to correlate with depth of invasion for patients suffering from cutaneous malignant melanoma.

## Materials and methods

Immunohistochemistry staining

Paraffin-embedded de-identified tissue microarrays (TMAs) were acquired from TissueArray.Com LLC (Derwood, MD, USA). The use of these tissue microarrays does not require IRB approval since the samples were de-identified by the providers and are not linked to any personal identifying information. These TMAs contained eight samples of normal skin and 83 samples of cutaneous melanoma with varying tumor, node, and distant metastasis (TNM) clinicopathological parameters and clinical stages. Throughout the process of immunohistochemistry (IHC) staining and computer-assisted image analysis, the experimenters were blinded to information related to the tissue samples. Antigen retrieval was done using a steamer, followed by a blocking step with 5% bovine serum albumin (BSA). Primary anti-PTEN antibody (catalog number 9559-138G6; Cell Signaling, Danvers, MA, USA) at 1:50 dilution was used to label the tissue samples, followed by incubation with a reciprocal secondary anti-rabbit immunoglobulin G (IgG) conjugated to horseradish peroxidase (HRP) (catalog number A9169; Sigma-Aldrich, St. Louis, MO, USA). Lastly, a 3’-diaminobenzidine (DAB) substrate was used to visualize the immunoreactivity within the tissue samples, followed by hematoxylin counterstaining and sealing with a coverslip for preservation.

Quantitative PTEN expression analysis

High-resolution images of the immunohistochemistry-stained tissue samples were acquired at 10× magnification using a Nikon A1R microscope (Nikon Instruments Inc., Melville, NY, USA). Aperio ImageScope software (Leica Biosystems Inc., Buffalo Grove, IL, USA) was used for computer-assisted image analysis to eliminate the human subjectivity associated with the manual scoring of the intensity of immunohistochemistry staining. A built-in positive pixel counting algorithm calculated the immunoreactivity scores in the regions of interest within each tissue sample. These manually outlined regions of interest included the normal epidermis or melanoma tissues and excluded the dermis or stroma in normal tissue samples and malignant melanoma samples, respectively. The algorithm then assigned red, orange, yellow, and blue colors to label strong positive pixels, medium positive pixels, weak positive pixels, and negative pixels, respectively. Immunoreactivity was determined based on the percentage of positive cells and their staining intensity. The histochemical score (H-score) was derived from the percentage of positive cells multiplied by the weighted staining intensity as follows: H-score = (1 × % weak positivity) + (2 × % medium positivity) + (3 × % strong positivity). Mean H-scores, as well as the standard error of the mean (SEM) values, were calculated for groups of cutaneous melanoma tissue samples based on their TNM clinicopathological parameters. Two-tailed unpaired T-tests were conducted for each comparison, setting the statistical significance at p ≤ 0.05.

## Results

Phosphatase and tensin homolog expression in the normal epidermis

**Figure 1:**
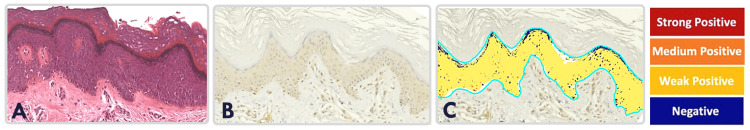
Phosphatase and tensin homolog expression in the normal epidermis. Representative normal skin tissue sample: (A) H&E stained; (B) IHC stained for PTEN immunoreactivity; (C) ImageScope analyzed (the epidermis is outlined and included in analysis while the dermis is excluded from analysis). H&E: hematoxylin and eosin; IHC: immunohistochemistry; PTEN: phosphatase and tensin homolog

Expression analysis for PTEN using immunohistochemistry showed a faint staining that was diffusely distributed in the normal epithelium of the epidermis (Figure 1).

Phosphatase and tensin homolog expression in cutaneous melanoma tumor samples

**Figure 2:**
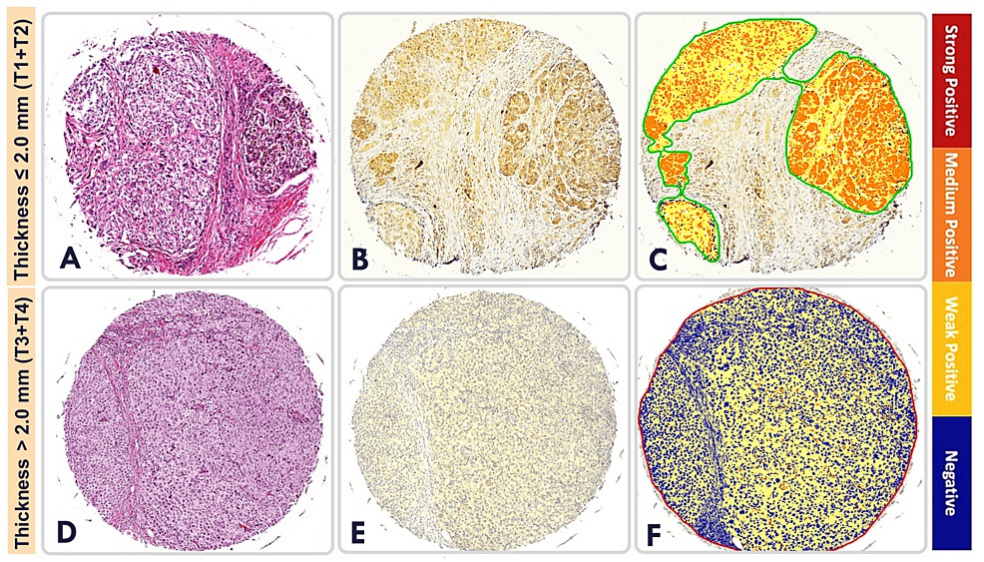
Phosphatase and tensin homolog expression in melanoma tissue samples with a thickness of ≤ 2.0 mm versus melanoma tissue samples > 2.0 mm Representative melanoma tissue sample with thickness ≤ 2.0 mm (T1+T2): (A) H&E stained; (B) IHC stained for PTEN immunoreactivity; (C) ImageScope analyzed (melanoma tissues are outlined and included in analysis while stroma is excluded from analysis). Representative melanoma tissue sample with thickness > 2.0 mm (T3+T4): (D) H&E stained; (E) IHC stained for PTEN immunoreactivity; (F) ImageScope analyzed. H&E: hematoxylin and eosin; IHC: immunohistochemistry; PTEN: phosphatase and tensin homolog

Melanoma tissue samples showed variations in PTEN expression. Specifically, the mean H-score for PTEN immunoreactivity in melanoma tumor samples with tumor thickness less than or equal to 2.0 mm (T1+T2) was 0.95, while the mean H-score for PTEN immunoreactivity was 0.57 in melanoma tumor samples with tumor thickness greater than 2.0 mm (T3+T4) (Figure 2).

**Figure 3:**
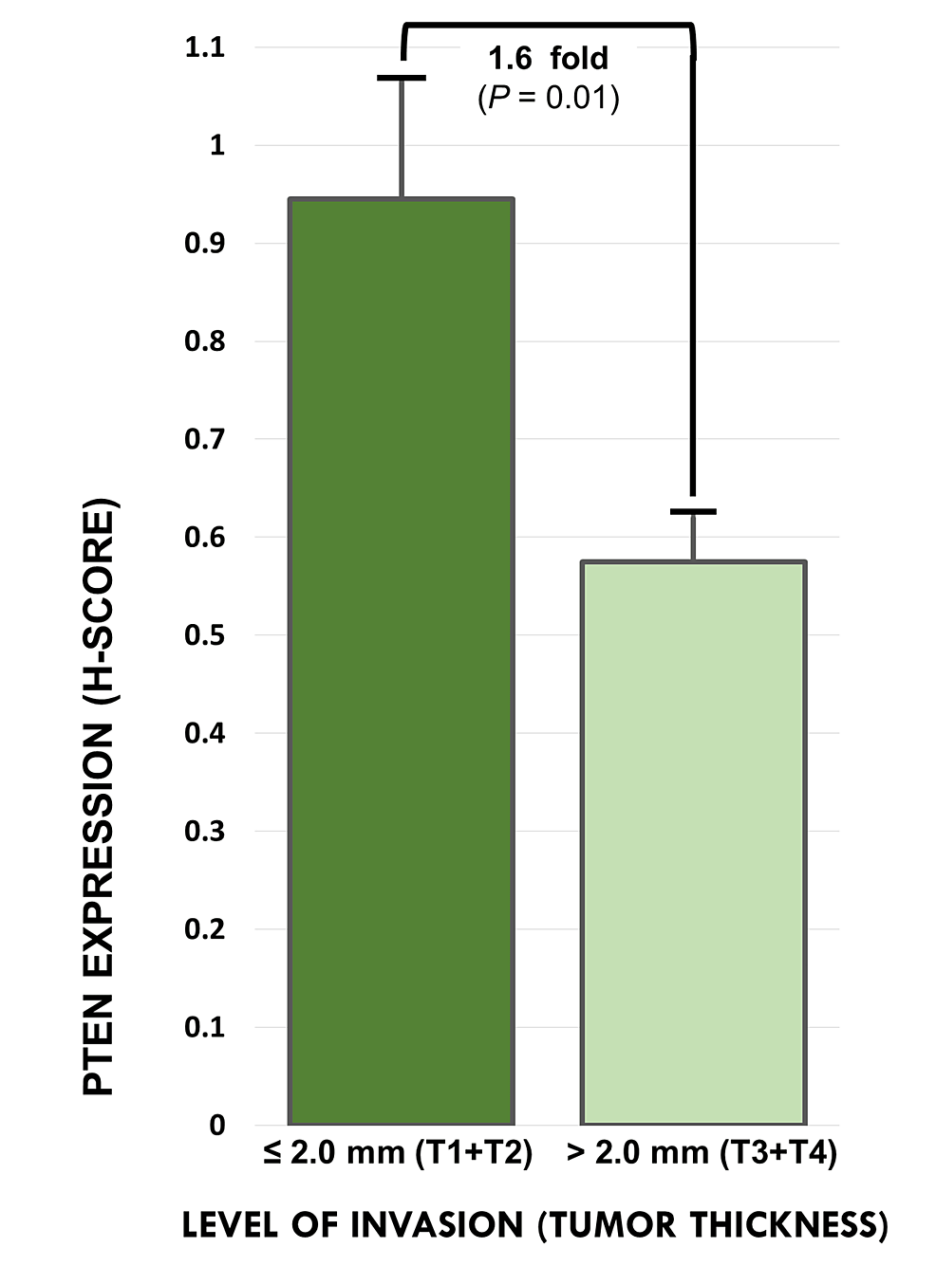
Phosphatase and tensin homolog expression as measured by mean H-scores in melanoma tissue samples ≤ 2.0 mm versus melanoma tissue samples > 2.0 mm A bar graph illustrating the mean H-score for PTEN immunoreactivity in melanoma tissue samples ≤ 2.0 mm (T1+T2) (N=8) versus > 2.0 mm (T3+T4) (N=75). H-score: histochemical score; PTEN: phosphatase and tensin homolog

Quantitative PTEN expression analysis, as measured by mean H-score, showed a 1.6-fold decrease in melanoma tissue samples with a thickness greater than 2.0 mm (N=75) compared to melanoma tissue samples with a tumor thickness less than or equal to 2.0 mm (N=8). This difference was statistically significant (p = 0.01) (Figure 3).

## Discussion

The tumor suppressor gene product, PTEN, is frequently inactivated in cancer cells, along with the loss of its ability to inhibit cancer cell growth and enhance cellular sensitivity to apoptosis [12, 13]. Specifically, the loss of PTEN surveillance leads to increased phosphorylation and activation of the AKR mouse transforming (AKT) serine/threonine kinase, also known as protein kinase B (PKB). The PI3K/AKT pathway is known to be highly active in primary melanoma [14], resulting in enhanced malignant tumor growth and inhibition of the apoptotic pathway in melanoma [15, 16]. Another consequence of PTEN loss, or downregulation, is the promotion of immune evasion and escape since immunogenic cell death is essential for the activation of tumor-specific cytotoxic T lymphocytes (CTL). Thus, this adds to the resistance to T-cell-mediated immunotherapies, such as immune checkpoint inhibitors, in these patients.

This study evaluated the utility of PTEN expression as a prognostic indicator for cutaneous melanoma. The PTEN expression was significantly downregulated in melanoma samples with a vertical tumor thickness greater than 2.0mm (T3+T4) when compared to melanoma samples with a vertical tumor thickness less than 2.0mm (T1+T2). This finding suggests the potential utility of PTEN protein expression as a surrogate biomarker to correlate with melanoma’s vertical growth, measured by tumor thickness, which has been shown to greatly affect survival rates in patients [17]. In fact, melanoma tumor thickness or depth, known as Breslow’s depth, is the best available prognostic predictor for survival [18]. Studies over the course of a 20-year period estimated the percentage of melanoma deaths with a measured thickness of less than 0.8 mm to be approximately 4.8% [19]. For tumors ranging from 0.8 mm to less than 1.0 mm, the percentage of melanoma deaths increased to 10.6% and generally increased to more than 30% for melanoma thickness greater than 3.0 mm [19]. Despite surveillance and early detection efforts in the United States, there has not been a less-than-optimal decline in the mortality rates in melanoma patients due to unfavorable prognoses in many patients. Therefore, it is crucial to continue to improve and implement new screening methods [20].

We envision that the quantitative analysis of PTEN expression in melanoma tissues may serve as a supplement to the histopathological examination to predict the aggressive behavior of a certain melanoma tumor, possibly even in the early stages of the disease, given that the vertical thickness of the tumor is associated with melanoma’s highly invasive and metastatic behaviors [21].

The restraints of this study primarily stem from the use of tissue microarrays comprising small tissue cores that may not accurately represent the entire tumor or the variability within it, despite the capability to process numerous tissue samples simultaneously, quickly, and consistently. Furthermore, because tissue microarray samples are usually archival, they do not provide real-time clinical data, including patients' medical histories, survival rates, and tumor recurrence rates.

Therefore, follow-up studies are needed to document PTEN expression patterns in a large cohort of cutaneous melanoma patients. The goal is to establish a correlation between PTEN protein expression levels and survival rate, or recurrence rate, in these patients in order to standardize PTEN as a biomarker that can supplement the current prognostic evaluation methods for patients with malignant melanoma.

## Conclusions

In this study, the expression of the PTEN tumor suppressor was significantly downregulated as the vertical tumor thickness of cutaneous melanoma increased. These findings suggest the potential utility of PTEN as a surrogate biomarker in the evaluation of cutaneous melanoma and the determination of individualized treatment plans for patients affected by malignant melanoma.
